# Recurrent Hematemesis From Downhill Esophageal Varices: A Therapeutic Challenge for Gastroenterologists

**DOI:** 10.7759/cureus.13840

**Published:** 2021-03-11

**Authors:** Hassam Ali, Shiza Sarfraz, Abeera Sarfraz

**Affiliations:** 1 Internal Medicine, East Carolina University, Vidant Medical Center, Greenville, USA; 2 Anesthesiology, Bahawal Victoria Hospital, Quaid-E-Azam Medical College, Bahawalpur, PAK; 3 Internal Medicine, Federal Medical and Dental College, Islamabad, PAK

**Keywords:** esrd, downhill varices, endoscopy, svc syndrome, end-stage renal disease

## Abstract

Downhill or upper esophageal varices have an etiology that differs from that of the “uphill” varices secondary to portal hypertension. Approximately 0.1% of all cases of variceal hemorrhage are due to downhill varices. The underlying etiology is obstruction of the superior vena cava (SVC) which results in the shunting of blood from the systemic circulation into the esophageal plexus, predominantly the upper two-thirds. The management should be directed to relieve the vascular obstruction. One of the causes of SVC obstruction leading to downhill variceal bleeding is dialysis catheter-associated SVC stenosis. We report the case of a 34-year-old male with hematemesis associated with downhill varices due to chronic SVC obstruction because of a central venous catheter.

## Introduction

Esophageal varices are usually associated with clinical conditions such as portal hypertension. Downhill or upper esophageal varices have been reported in superior vena cava (SVC) obstruction due to the mass effect of the tumor [1,2]. End-stage renal disease (ESRD) patients on dialysis can develop downhill varices because of central venous catheter-related SVC syndrome [2-4]. Additionally, some unique mechanisms of downhill esophageal varices include Behçet’s disease, retrosternal goiter, and severe pulmonary hypertension. Treatment of downhill varices is dependent upon initial presentation and underlying etiology. We describe the case of an ESRD patient with hemorrhagic downhill varices limited to the upper esophagus, attributed to chronic SVC syndrome because of a central venous catheter. ESRD itself can contribute to increased bleeding risk owing to regular anticoagulants used in dialysis sessions and uremic coagulation dysfunctions.

## Case presentation

A 34-year-old male presented to the emergency department with complaints of two episodes of hematemesis within the last three days. The hematemesis was accompanied by epigastric pain, fatigue, headache, and melena. The patient had a significant previous medical history for hypertension, ESRD on hemodialysis, SVC syndrome, and upper gastrointestinal (GI) bleeds secondary to peptic ulcer disease. Previously, the patient was admitted multiple times for similar complaints, and the last esophagogastroduodenoscopy (EGD) revealed proximal and middle esophageal varices in the absence of lower esophageal varices, and he was treated with endoscopic band ligation. Previously, he had refused to undergo further investigations.

**Figure 1:**
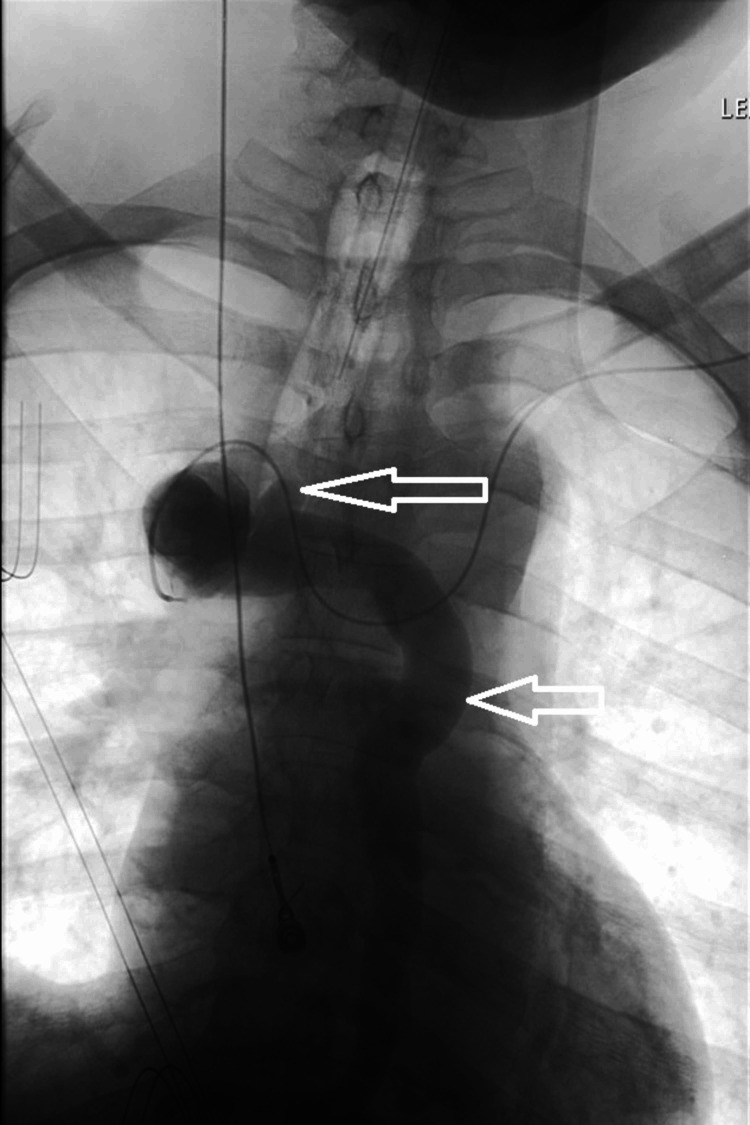
Superior venacavogram showing SVC occlusion (white arrows). SVC = superior vena cava

On presentation, his vital signs were stable. His physical examination was positive for epigastric and left lower quadrant bowel tenderness. The rest of the examination was non-focal. His hemoglobin was 7.5 g/dL from his baseline of 11 g/dL with a low hematocrit. Prothrombin time and international normalized ratio were within normal limits. The patient underwent endoscopy, which was significant for grade III downhill varices in the upper third of the esophagus with a positive red wale sign. The patient also had medium size varices in the mid distal third of the esophagus. Vascular interventional radiology was consulted, and a superior venacavogram was done, which demonstrated chronic SVC occlusion without a residual lumen or channel (Figure 1). The decision was made to consult cardiothoracic surgery and decompress the central venous system’s high pressure by bypass surgery. It was proposed that this will reduce the risk of further variceal bleeding. The patient underwent SVC to right atrium bypass using a 24-mm aortic homograft. The patient was subsequently discharged and followed up on an outpatient basis. He did not have further episodes of hematemesis.

## Discussion

Downhill varices are referred to as retrograde blood flow in the veins. The blood from the upper two-thirds of the esophagus is drained to the SVC by multiple tributaries. The lower third drains in the left gastric vein and through that into the portal vein. In the obstruction in SVC syndrome, the blood from the upper two-thirds of the esophagus drains to the right atrium through several collaterals. This also includes venous drainage into the azygous/hemiazygos system, which then drains into the patent part of the SVC. This retrograde blood flow can result in upper esophageal varices. These are often called downhill varices because the blood flow is opposite to that of uphill varices, as seen in liver disease [1]. The diversion of blood along the peri-esophageal plexus and the portal vein can extend with prolonged obstruction [2]. When associated with downhill varices, SVC syndrome is typically caused by surrounding malignancies, resulting in mass effect and compression. Dialysis catheters can also result in SVC obstruction. The pathophysiology is derived from possible endothelial injury. Some hypothesized factors include initial trauma during line placement, foreign body in the vessel resulting in turbulent blood flow, and localized injury due to catheter movement during respiration or movement. All these factors can result in physical injury to the vessel resulting in an inflammatory response [3]. Platelet aggregation can result in the activation of leukocytes and myeloperoxidase release and profibrotic cytokines, contributing to intravascular thrombosis. Another proposed etiology for SVC syndrome due to dialysis-related catheter is intimal hyperplasia caused by turbulence [3].

Downhill esophageal varices are less likely to bleed than uphill varices seen in liver cirrhosis [4]. Most of these varices are recognized after an episode of hematemesis [1]. The reduced risk of bleeding could be due to the downhill varices being under submucosa in the upper two-thirds of the esophagus compared to the more superficial location of uphill varices on the lower third of the esophagus. Additional risks that increase the chances of bleeding uphill varices are the underlying conditions. For example, cirrhosis, one of the prime causes of uphill varices, also results in coagulopathies and can increase the risk of bleeding. However, ESRD patients on dialysis developing SVC syndrome due to catheters theoretically have an increased risk of bleeding if they develop downhill varices compared to other etiologies such as malignancies. This is due to regular anticoagulants used in dialysis sessions and uremic coagulation dysfunctions [5].

Most clinical presentations, resulting in GI bleed, require endoscopy for bleeding control. Occlusion can be diagnosed via computed tomography angiogram (CTA) and or magnetic resonance venography (MRV). Our patient already had an established SVC occlusion and had refused any interventional procedures in the past. The best treatment recommendations point towards decompression of hypertension by relieving the obstruction [6,7]. In SVC occlusion, percutaneous transluminal angioplasty with or without stenting is a viable option, although some patients should undergo bypass surgery if that is not an option [8]. Our patient’s chronic occlusion was more likely to respond to bypass surgery, and there were no further episodes of hematemesis after his bypass. He did not undergo a follow-up endoscopy to see the resolution of downhill varices, although we believe that would not have changed the prognosis. Patients with upper variceal bleeding, who have a history of ESRD and central venous catheters on hemodialysis, should be evaluated for downhill varices and SVC syndrome. Imaging studies can include venography, MRV, or CTA. No data suggest surveillance for downhill varices in dialysis in patients exhibiting venous occlusion due to catheters. Additional causes of downhill varices may include retrosternal goiter, Behçet’s disease, and pulmonary hypertension. Our case highlights the importance of having a high clinical suspicion so that early recognition and treatment can help prevent significant morbidity.

## Conclusions

This case highlights an unusual location and etiology of downhill bleeding varices, which requires a unique approach for management. In the case of SVC occlusion secondary to venous catheters, although endoscopy with banding can be done as a temporizing measure for active bleeding, relief of the underlying SVC obstruction is the cornerstone for definitive treatment of downhill varices. ESRD patients can be at an increased risk of downhill variceal bleed due to dialysis catheter-associated SVC obstruction.
